# Identification of Functional Amino Acid Residues Involved in Polyamine and Agmatine Transport by Human Organic Cation Transporter 2

**DOI:** 10.1371/journal.pone.0102234

**Published:** 2014-07-14

**Authors:** Kyohei Higashi, Masataka Imamura, Satoshi Fudo, Takeshi Uemura, Ryotaro Saiki, Tyuji Hoshino, Toshihiko Toida, Keiko Kashiwagi, Kazuei Igarashi

**Affiliations:** 1 Graduate School of Pharmaceutical Sciences, Chiba University, Chiba, Japan; 2 Amine Pharma Research Institute, Innovation Plaza at Chiba University, Chiba, Japan; 3 Faculty of Pharmacy, Chiba Institute of Science, Chiba, Japan; ENEA, Italy

## Abstract

Polyamine (putrescine, spermidine and spermine) and agmatine uptake by the human organic cation transporter 2 (hOCT2) was studied using HEK293 cells transfected with pCMV6-XL4/hOCT2. The *Km* values for putrescine and spermidine were 7.50 and 6.76 mM, and the *Vmax* values were 4.71 and 2.34 nmol/min/mg protein, respectively. Spermine uptake by hOCT2 was not observed at pH 7.4, although it inhibited both putrescine and spermidine uptake. Agmatine was also taken up by hOCT2, with *Km* value: 3.27 mM and a *Vmax* value of 3.14 nmol/min/mg protein. Amino acid residues involved in putrescine, agmatine and spermidine uptake by hOCT2 were Asp^427^, Glu^448^, Glu^456^, Asp^475^, and Glu^516^. In addition, Glu^524^ and Glu^530^ were involved in putrescine and spermidine uptake activity, and Glu^528^ and Glu^540^ were weakly involved in putrescine uptake activity. Furthermore, Asp^551^ was also involved in the recognition of spermidine. These results indicate that the recognition sites for putrescine, agmatine and spermidine on hOCT2 strongly overlap, consistent with the observation that the three amines are transported with similar affinity and velocity. A model of spermidine binding to hOCT2 was constructed based on the functional amino acid residues.

## Introduction

Polyamines (putrescine, spermidine and spermine) are present at millimolar concentrations in both prokaryotic and eukaryotic cells and play important roles in cell growth and differentiation [1], [2]. The polyamine content in cells is regulated by biosynthesis, degradation and transport [1], [3]. In *Escherichia coli*, we thus far reported the properties of several polyamine transport systems [3]–[6]. Those are spermidine-preferential and putrescine specific uptake systems as well as PotE (involved in the excretion of putrescine by a putrescine-ornithine antiporter activity) and CadB (involved in the excretion of cadaverine by a cadaverine-lysine antiporter activity). The former two transport systems function at neutral pH to maintain the optimal concentrations of putrescine and spermidine [7], whereas the latter two transport systems function at acidic pH to neutralize the external microenvironment and to generate a proton motive force [8]. A spermidine excretion system (MdtJI) also functions when spermidine over-accumulates in cells [9]. Furthermore, it has been reported that PuuP functions as a putrescine transporter when putrescine is used as an energy source under glucose starvation [10], [11]. In this case, it was necessary to accumulate high concentrations of putrescine in cells [11]. In *Saccharomyces cerevisiae*, there are four kinds of polyamine uptake proteins (DUR3, SAM3, GAP1 and AGP2), containing either 12 or 16 transmembrane segments [12]–[14]. Of these, DUR3 and SAM3 are the main contributors to polyamine uptake. There are also five kinds of polyamine excretion proteins (TPO1–5), consisting of 12 transmembrane segments, to maintain the optimal concentrations of polyamines [15], [16]. Among them, TPO1 and TPO5 were the most active proteins. Since the polyamine metabolizing enzyme spermidine/spermine *N* ^1^-acetyltransferase is not present in yeast, this likely explains why five kinds of excretion proteins are present.

Furthermore, the existence of putrescine and spermidine transport protein LmPOT1 in *Leishmania major* [17], putrescine and cadaverine transport proteins TcPOT1.1 and TcPOT1.2 in *Trypanosoma cruzi* [18], the putrescine, spermidine and spermine transporter encoded by the RMV1 gene in *Arabidopsis thaliana* [19] and carnitine and spermidine transporter SLC22A16 in NT2/D1 human testicular cancer cells [20] has been reported. It has been also reported that putrescine and agmatine are taken up by human OCT2 (SLC22A2) [21], and spermidine is by mouse OCT2 [22]. In this communication, we studied the properties of putrescine, agmatine and spermidine uptake by hOCT2 in detail, and found that the active center of putrescine, agmatine and spermidine uptake are located on α-helices 9 to 12. Amino acid residues on α-helices 9 to 12 involved in the uptake of the three amines were identified, and modeling of polyamine binding to hOCT2 was carried out based on the identification of functional amino acids.

## Materials and Methods

### Plasmids

The pCMV6-XL4 plasmid (Origene Technologies) containing hOCT1, hOCT2, hOCT2-A, and hOCT3 cDNA [23], [24], and the pcDNA3.1(+) (Life Technologies) containing hMATE2-K cDNA [25] were kindly provided by Drs. K. Inui and S. Masuda (Kyoto University Hospital, Kyoto, Japan). Plasmids pcDNA3 (Life Technologies), containing OCTN1 [26], OCTN2 [27] and hMATE1 cDNA [28] were kindly provided by Dr. A. Tsuji (Kanazawa University, Kanazawa, Japan) and Y. Moriyama (Okayama University, Okayama, Japan), respectively.

### Cell Culture and Transfection

HEK293 cells (ATCC CRL-1573, American Type Culture Collection) were cultured in Dulbecco's modified Eagle's medium with 10% fetal bovine serum, 100 units/ml penicillin G and 50 units/ml streptomycin in an atmosphere of 5% CO_2_/95% air at 37°C. Plasmids were purified using a QIAGEN plasmid Midi kit (QIAGEN GmbH) according to the manufacturer's instructions. HEK293 cells (1×10^6^ cells) were cultured on poly-D-lysine coated 6-well plates for 24 h, and then transfection (2 µg of plasmid cDNA per well) was performed with Lipofectamine 2000 (Life Technologies) according to the manufacturer's instructions. After 24 h culture, the cells were used for polyamine transport experiments.

### Polyamine Transport Assay

HEK293 cells (1×10^6^ cells) in 0.99 ml of NaCl buffer (135 mM NaCl, 1 mM MgCl_2_, 2 mM CaCl_2_, 10 mM glucose, and 20 mM Hepes-NaOH, pH 7.4) were incubated at 37°C for 10 min, and then the uptake assay was started by the addition of 10 µl of 50 mM each of [^14^C]putrescine (18.5 MBq/mmol), [^14^C]agmatine (18.5 MBq/mmol), [^14^C]spermidine (37 MBq/mmol) or [^14^C]spermine (37 MBq/mmol), respectively. After incubation at 37°C for 30 min, cells were washed 2 times with 2 ml of ice-cold NaCl buffer containing 5 mM each of putrescine, agmatine, spermidine or spermine. Washed cells were treated with 0.2 ml of 5% TCA and then lysed with 0.2 ml of 0.2 M NaOH, and a 0.3 ml aliquot was used for the measurement of radioactivity by a liquid scintillation counter. When substrate specificity was tested, putrescine, agmatine, spermidine, spermine, γ-aminobutyric acid (GABA), ornithine, lysine, arginine and histidine were added to the reaction mixture at a 20-fold higher concentration than substrate. Protein content was determined by the method of Lowry et al. [29].

### Assay for Tetraethylammonium (TEA) and Carnitine Transport

This was performed as described above using 0.5 mM [ethyl-1-^14^C]tetraethylammonium bromide (18.5 MBq/mmol) and 2 µM [methyl-^3^H]carnitine hydrochloride (2.03 GBq/mmol) as substrate.

### Site-Directed Mutagenesis

For preparation of pcDNA3.1/hOCT2-*myc*-His, PCR was performed using hOCT2 cDNA as template and 5′-CCTGGATCCAGGATCATGCCCACCACCGTGGAC-3′ (P1) and 5′-CTCTCTAGAGTTCAATGGAATGTCTAGTTTCTG-3′ (P2) as primers. The PCR products thus obtained were digested with XbaI and BamHI, and the fragments were inserted into the same restriction site of pcDNA3.1/*myc*-His A (Life Technologies). Site-directed mutagenesis of Asp to Asn, Glu to Gln on hOCT2 cDNA was carried out with the QuickChange Site-directed mutagenesis kit (Stratagene). A list of oligonucleotide primers used for mutagenesis was shown in Table S1. Mutations were confirmed by DNA sequencing using a CEQ8000 DNA genetic analysis system (Beckman Coulter).

### Western Blot Analysis

Expression level of hOCT2-*myc*-His and its mutated proteins on plasma membrane was measured using 2×10^6^ cells transfected with pcDNA3.1/hOCT2-*myc*-His and Pierce Cell Surface Protein Isolation Kit (Thermo Scientific) according to the manufacturer's protocol using rabbit anti-6His antibody (BETHYL Laboratories). Briefly, cell surface proteins were labelled with membrane-impermeable Sulfo-NHS-SS-Biotin reagent, and the treated cells were lysed. After the labelled membrane proteins were isolated with immobilized NeutrAvidin Agarose according to the manufacturer's instruction, the membrane proteins were eluted with SDS-PAGE sample buffer containing 50 mM dithiothreitol. Eluted proteins were analyzed by Western blotting after separation with 10.5% SDS-PAGE.

### Construction of a Model of the Secondary Structure of hOCT2 and hOCT2-A

hOCT2 consists of 555 amino acids and is predicted to have 12 α-helical transmembrane segments, an intracellular N-terminus, a large glycosylated extracellular loop between transmembrane segments 1 and 2, and an intracellular C-terminus [30], [31]. In contrast, hOCT2-A, a splicing variant of hOCT2, consists of 483 amino acids and was predicted to have 9 α-helical transmembrane segments. Since the difference of amino acid sequence between hOCT2 and hOCT2-A was observed at the COOH terminal region, a putative topology of the COOH terminal region (57 amino acids) in hOCT2-A was determined using the transmembrane hidden Markov model (TMHMM) [32].

### Docking Simulation of Spermidine to hOCT2

Homology model of hOCT2 was constructed according to the method of Zhang et al. [30] based on the crystal structure of the glycerol 3-phosphate transporter of *Escherichia coli* GlpT (Protein Bank code 1pw4). Relative position of α-helices 1 to 12 was constructed accordingly. Atom coordinates of hOCT2 were kindly offered by Dr. S. H. Wright, University of Arizona, USA. The binding mode of spermidine to hOCT2 was predicted by docking simulations using GOLD ver. 5.2 [33]. Binding score was calculated to evaluate the binding affinity of spermidine to hOCT2. In the present docking simulation, the search area for docking was restricted within 40 Å from Cγ atom of Asp^475^, because there is an adequate space at Asp^475^ for spermidine to be bound to. Ten binding poses were generated for the spermidine binding. The binding affinities of those binding poses were estimated by the Gold score function. Based on the ranking in the estimated Gold score, the most probable binding pose was selected. Binding affinity for the selected binding pose was re-estimated using the Chem score function. This two step approach; i.e., the determination of binding pose with Gold score and the subsequent estimation of binding affinity with Chem score, was used for reliable prediction in docking simulations of low molecular weight molecules to a target enzyme [34]. The Gold score calculated for spermidine binding was 48.50, and the Chem score for spermidine was 19.97, respectively.

### Statistics

Values are indicated as mean ± S.E. Data were analyzed by Student's *t* test, and a statistical difference was shown by probability values.

## Results

### Polyamine and Agmatine Uptake Activities by hOCTs and hMATEs

**Figure 1:**
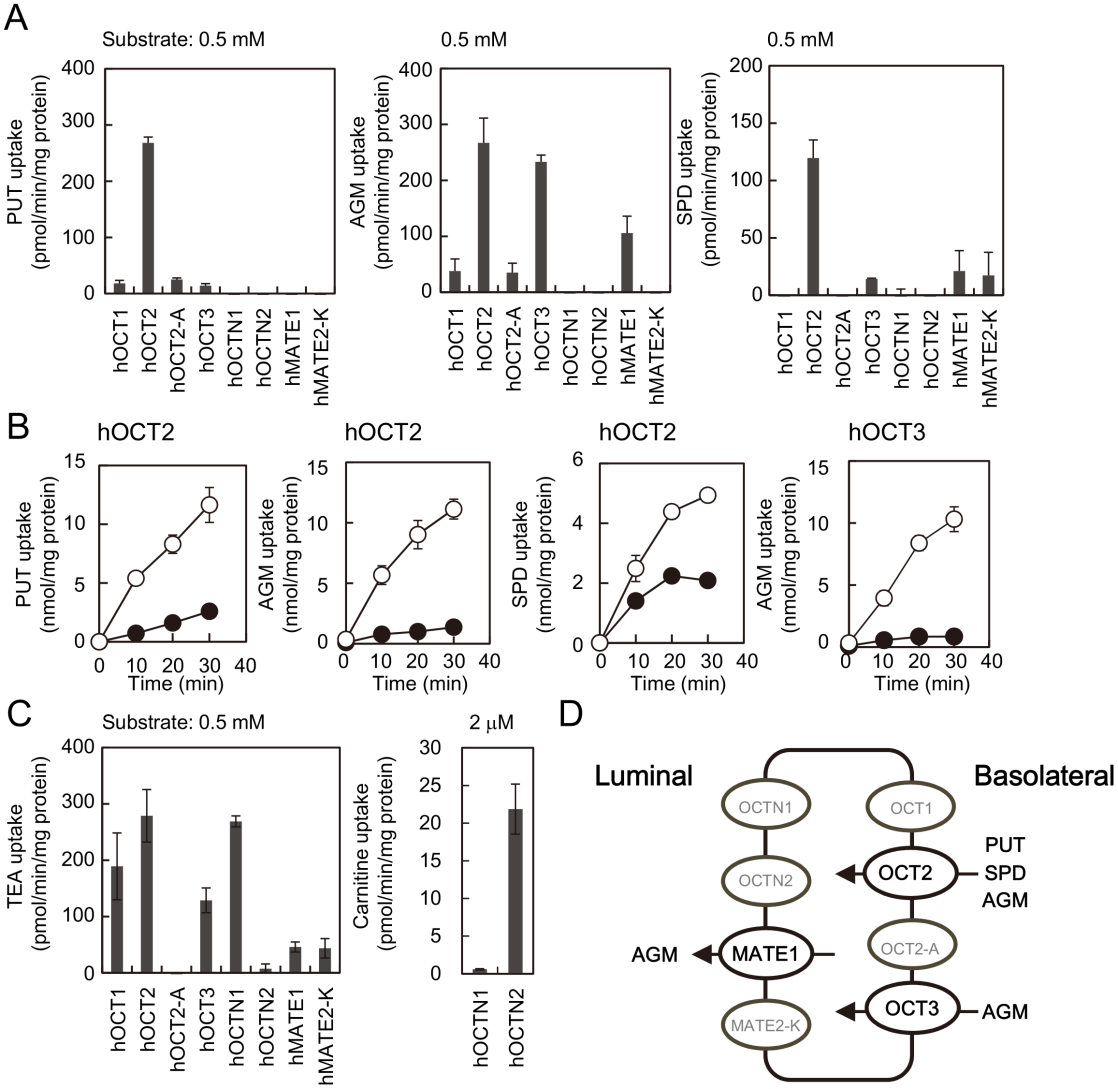
Polyamine, agmatine, TEA and carnitine uptake activities by various organic cation transporters expressed in HEK293 cells. (A) Polyamine and agmatine transport activities of HEK293 cells transfected with pCMV-XL4, pcDNA3.1(+) or pcDNA3 including a gene for hOCT or hMATE were measured as described in Materials and Methods. Activity of HEK293 cells transfected with the vector only was subtracted from the activity of HEK293 cells transfected with hOCT1, hOCT2, hOCT2-A, hOCT3, hOCTN1, hOCTN2, hMATE1 or hMATE2-K cDNA. (B) Time course of polyamine and agmatine uptake by hOCT2 or hOCT3. ○, pCMV6-XL4 plasmid containing hOCT2 or hOCT3 cDNA; •, pCMV6-XL4 vector. Data shown are the mean ± S.E. of triplicate determinations. (C) TEA and carnitine uptake activities by various organic cation transporters were measured as described in Materials and Methods. (D) Presence of hOCTs and hMATEs in kidney [35] and their polyamine and agmatine uptake activities are summarized.

**Figure 2:**
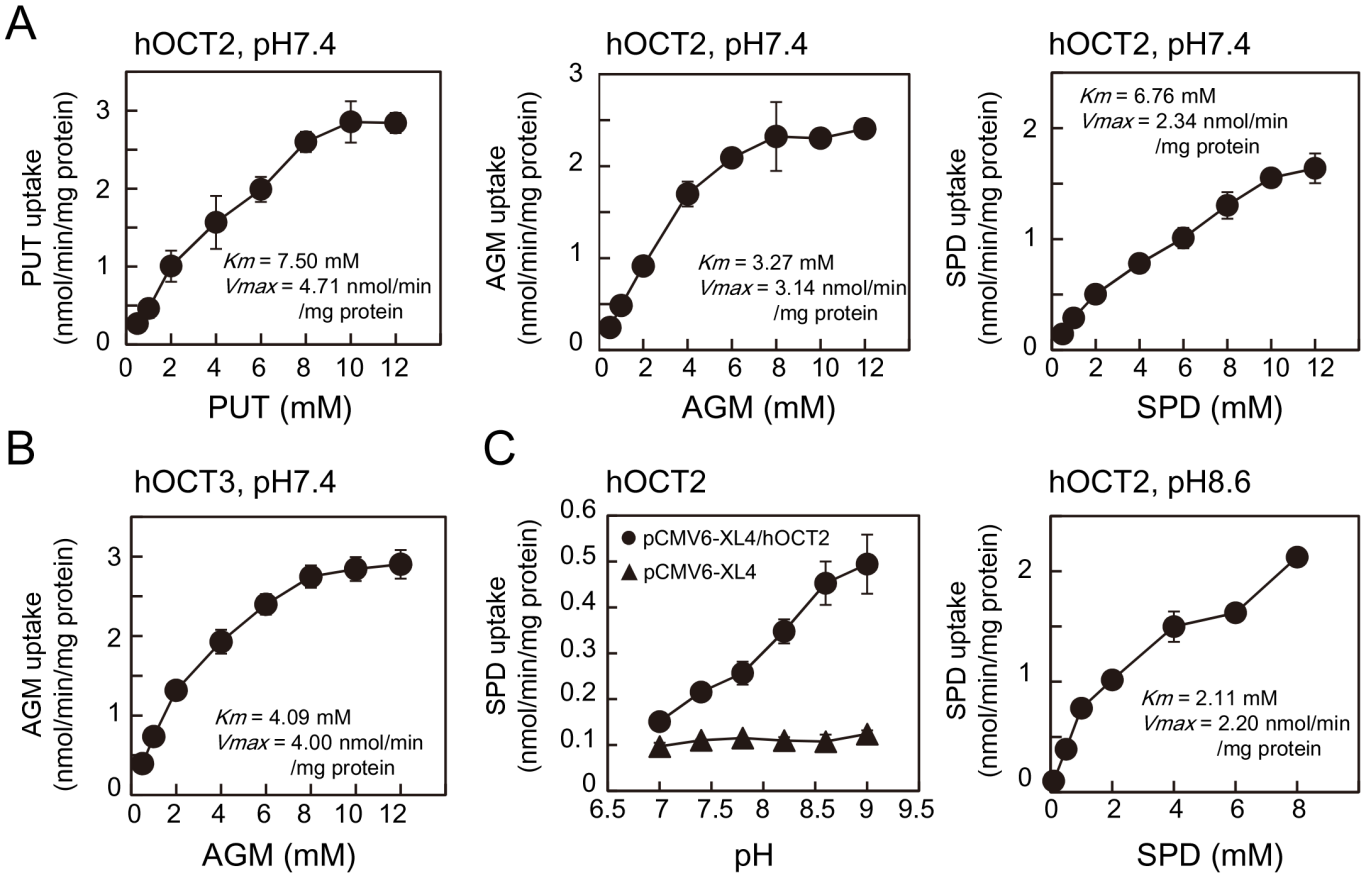
Determination of the *Km* and *Vmax* values of polyamine and agmatine uptake by hOCT2 and hOCT3. (A and B) Polyamine and agmatine uptake activities were measured as described in the legend of Fig. 1 using various concentrations of substrates. The *Km* and *Vmax* values for putrescine, agmatine and spermidine by hOCT2 and hOCT3 determined by Lineweaver-Burk plot are also shown. (C) Spermidine uptake by hOCT2 was measured at various pHs, and the *Km* and *Vmax* values were calculated at pH 8.6.

First, putrescine, agmatine and spermidine uptake by six hOCTs (organic cation transporters) and two hMATEs (multidrug and toxic compound extrusion proteins) normally found in human kidney (Figure 1D) [30], [35] were examined using HEK293 cells transfected with a cDNA encoding hOCTs or hMATEs. As shown in Figure 1A, significant uptake of putrescine, agmatine and spermidine by hOCT2 was observed. In addition, hOCT3 and hMATE1 catalyzed agmatine uptake. Among these activities, agmatine uptake by hOCT3 is a new finding in this study. The rate of uptake by hOCT2 was in the order agmatine ≈ putrescine > spermidine in the presence of 0.5 mM agmatine, putrescine or spermidine as a substrate (Figure 1A and 1B). The *Km* values for putrescine, agmatine, and spermidine uptake by hOCT2 were 7.50, 3.27 and 6.76 mM, respectively, and the *Vmax* values were 4.71, 3.14 and 2.34 nmol/min/mg protein, respectively (Figure 2A). Values for the *Km* and *Vmax* of agmatine uptake at hOCT3 were 4.09 mM and 4.00 nmol/min/mg protein, respectively (Figure 2B). Spermine uptake was not observed at pH 7.4 with any hOCTs and hMATEs tested (data not shown). Expression of plasmid-dependent other hOCTs and hMATEs was confirmed by measuring TEA and carnitine uptake activities (Fig. 1C). Expression of hOCT2-A, a splicing variant of hOCT2, was confirmed by measuring its mRNA level (data not shown).

Since diamines were good substrates for hOCT2, it was expected that spermidine might be taken up at alkaline pH. As shown in Figure 2C, the maximal spermidine uptake was observed at pH 8.6. The *Km* and *Vmax* values for spermidine uptake at pH 8.6 were 2.11 mM and 2.20 nmol/min/mg protein, respectively. The results indicate that the increase in spermidine uptake at pH 8.6 is probably due to a decrease in the *Km* value.

**Figure 3:**
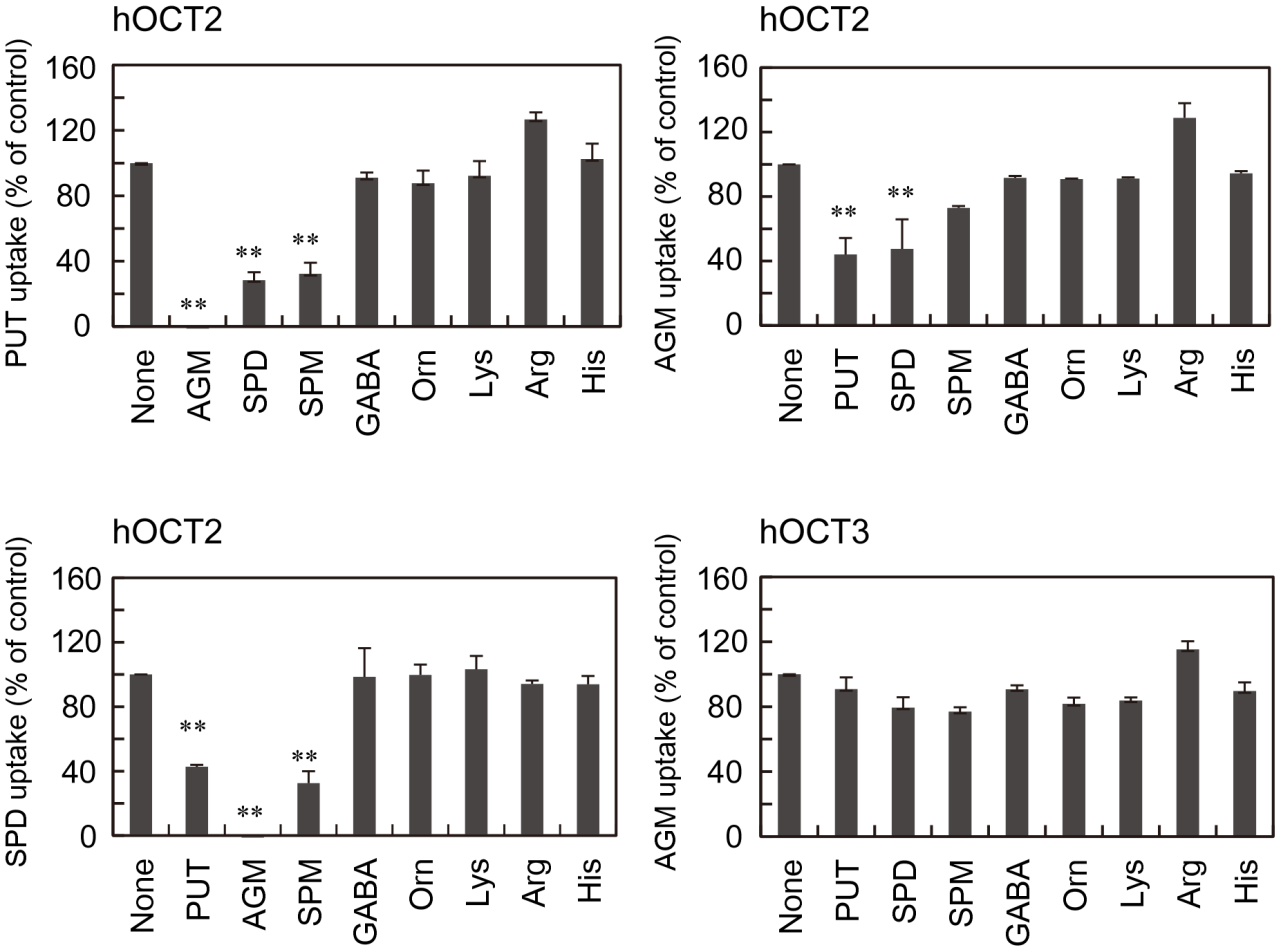
Effect of non-labeled polyamines, agmatine, γ-aminobutyric acid (GABA) and several amino acids on polyamine and agmatine uptake by hOCT2 and hOCT3. Polyamine and agmatine transport activities were measured in the presence of a 20-fold concentration (10 mM) of non-labeled polyamines, agmatine, GABA and several amino acids, and are shown as percent activity of control (None). Putrescine, agmatine and spermidine uptake activity by HEK293 cells transfected with pCMV6-XL4/hOCT2 was 258, 257, 128 pmol/min/mg protein, respectively in the presence of 0.5 mM [^14^C]putrescine, [^14^C]agmatine or [^14^C]spermidne as the substrate. Agmatine uptake by hOCT3 was 223 pmol/min/mg protein. Data shown are the mean ± S.E. of triplicate determinations. *p<0.05; **p<0.01.

Substrate specificity of hOCT2 and hOCT3 was studied by adding a 20-fold concentration of unlabeled polyamines, agmatine, γ-aminobutyric acid (GABA) and several amino acids (Figure 3). Putrescine uptake by hOCT2 was inhibited strongly by agmatine, and then by spermidine and spermine. The results confirm that the affinity for agmatine of hOCT2 is stronger than that for putrescine. Agmatine uptake by hOCT2 was inhibited by putrescine and spermidine to a similar degree. Spermidine uptake was inhibited in the order agmatine > spermine > putrescine. These results suggest that the putrescine binding site on hOCT2 may overlap with the agmatine and spermidine binding site. Agmatine uptake by hOCT3 was not inhibited by the three polyamines and basic amino acids (Figure 3), suggesting that agmatine is preferentially recognized by hOCT3.

### Identification of Amino Acid Residues Involved in Putrescine, Agmatine and Spermidine Uptake Activities in hOCT2

**Figure 4:**
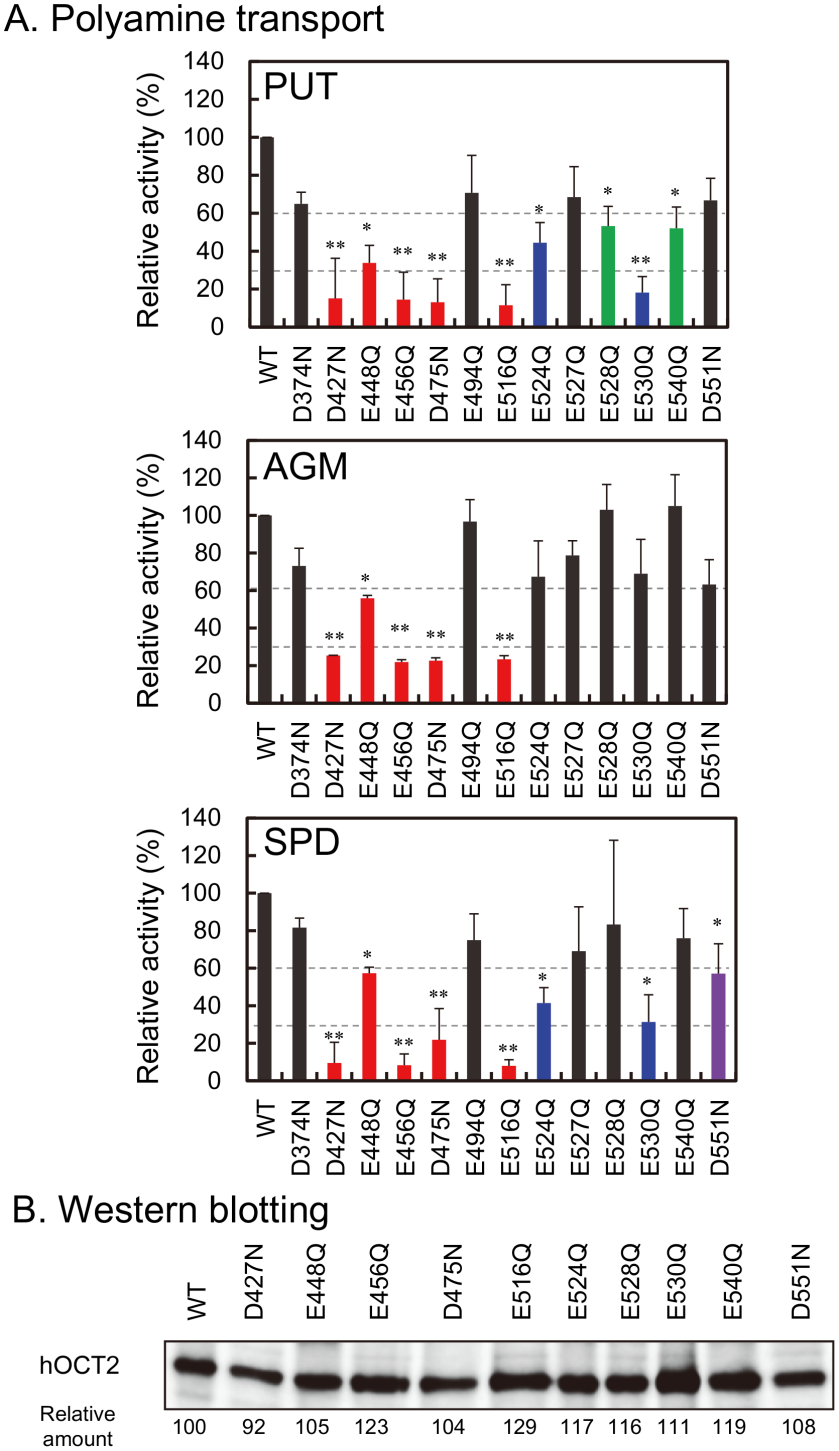
Identification of amino acid residues in hOCT2 involved in putrescine, agmatine and spermidine uptake by site-directed mutagenesis. (A) Putrescine, agmatine and spermidine uptake activities of hOCT2-*myc*-His mutants. Asp and Glu in hOCT2 were mutated to Asn and Gln, respectively. Putrescine, agmatine and spermidine uptake activities of the mutants are shown as percent activity of control (WT). Putrescine, agmatine and spermidine uptake by HEK293 cells transfected with pcDNA3.1/hOCT2-*myc*-His were 202, 205 and 120 pmol/min/mg protein, respectively. Values are means ± S.E. of triplicate determinations. *p<0.05; **p<0.01. (B) Expression level of hOCT2-*myc*-His mutant proteins in HEK293 cells. Western blot analysis was performed as described in the Materials and Methods. Relative amount of the expression level of hOCT2-*myc*-His mutant proteins on plasma membrane is shown as mean of triplicate determinations.

**Figure 5:**
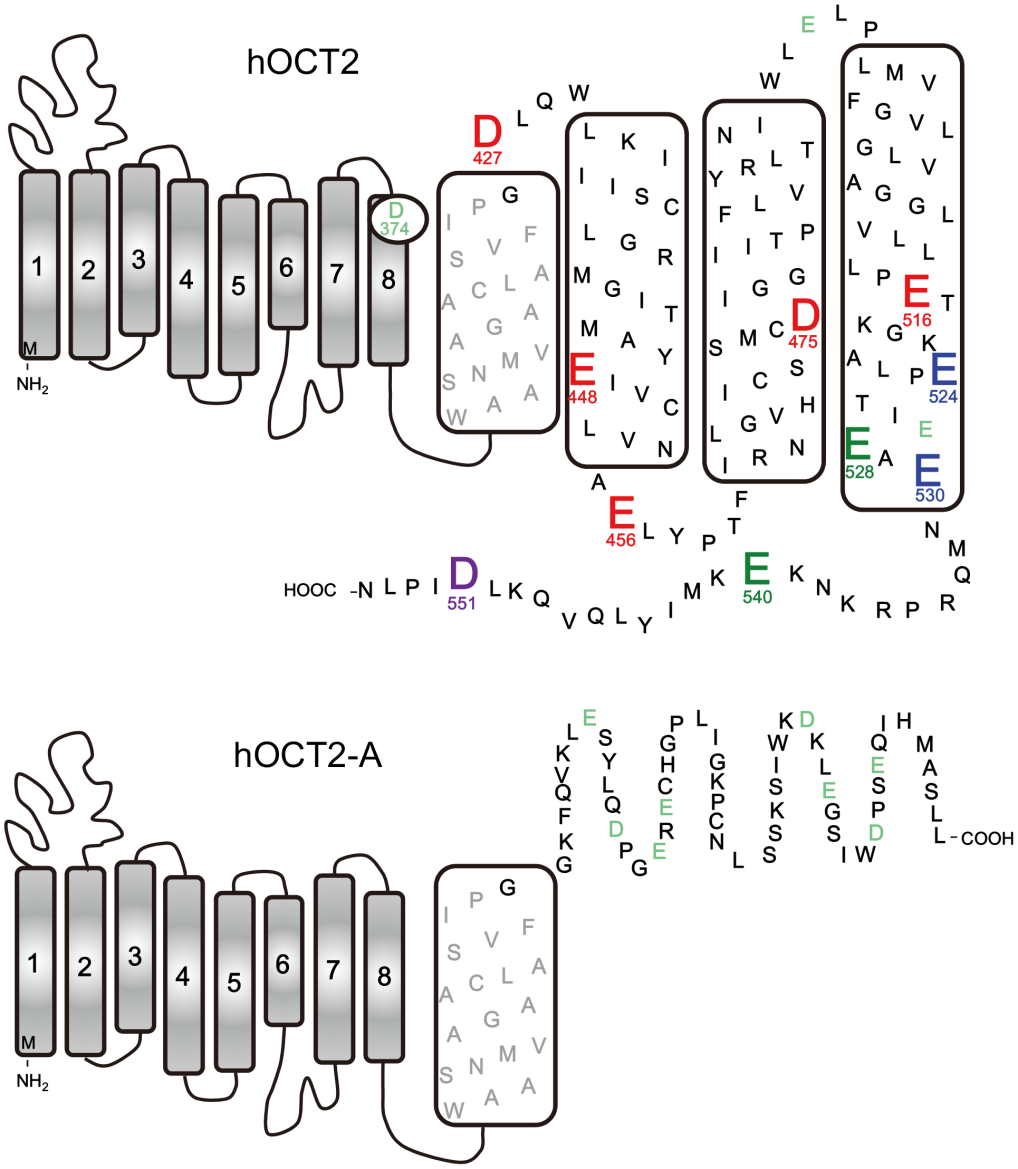
Functional acidic amino acid residues in hOCT2. Putative secondary structure models of hOCT2 and hOCT2-A are shown. Functional acidic amino acid residues in hOCT2 are shown in red, blue, green and violet. Asp^427^, Glu^448^, Glu^456^, Asp^475^ and Glu^516^ were involved in putrescine, agmatine and spermidine uptake activity (red), and Glu^524^ and Glu^530^ in putrescine and spermidine uptake (blue). Glu^528^ and Glu^540^ were involved in putrescine uptake activity (green), and Asp^551^ in spermidine uptake activity (violet).

**Figure 6:**
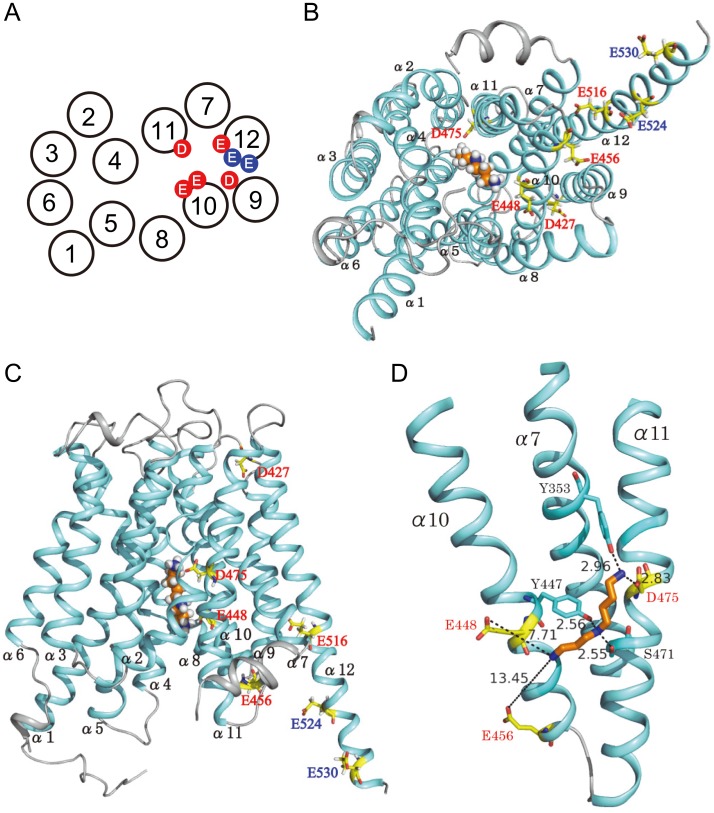
Modeling of spermidine binding to hOCT2. (A) Amino acid residues involved in transport activity of the three amines (red) and of putrescine and spermidine (blue) are shown on α-helices 1 to 12, whose relative positions were constructed based on the structure model of hOCT2 [31]. (B) View of the model from the cytoplasmic aspect of hOCT2. The 12 α-helices (blue) of hOCT2, and spermidine (white, proton; orange, carbon; blue, nitrogen) bound to hOCT2 are shown. (C and D) Side view of the model, with the cytoplasmic aspect of the protein directed toward the bottom. Spermidine binding site on hOCT2 is shown in more detail together with the functional amino acids in D. The distance (Å) between the amino acid residues involved in the interaction with spermidine and the primary amino- or secondary amine-group of spermidine is shown.

It has been reported that acidic amino acids are involved in the recognition of amino groups in polyamines [6]. Since hOCT2-A, a splicing variant of hOCT2, did not show significant putrescine, spermidine and agmatine uptake (see Figure 1), acidic amino acids present in the COOH terminal side after α-helix 9 in hOCT2 were mutated to neutral amino acids, i.e. Asp to Asn and Glu to Gln (see Figure 4), and putrescine, agmatine and spermidine uptake activities were evaluated. As shown in Figure 4A, Asp^427^, Glu^448^, Glu^456^, Asp^475^ and Glu^516^ were important for uptake of the three amines. These acidic amino acid residues may interact with the protonated amino groups of putrescine, agmatine and spermidine. In addition to the five acidic amino acids, Glu^524^ and Glu^530^ were involved in putrescine and spermidine uptake, and Glu^528^ and Glu^540^ were weakly involved in putrescine uptake activity. Furthermore, Asp^551^ was also involved in the recognition of spermidine. As a control, Asp^374^ on α-helix 8 was mutated to Asn, but uptake activities of the three amines did not change significantly (Figure 4A). Under these conditions, the amount of hOCT2 mutants on plasma membrane was evaluated. As shown in Figure 4B, the level of hOCT2 mutants on plasma membrane was nearly equal, confirming that these amino acid residues are involved in the recognition of substrates. These results indicate that recognition sites of putrescine, agmatine and spermidine on hOCT2 strongly overlap, concordant with the finding that the three amines are transported with similar affinity and velocity. The location of acidic amino acid residues involved in putrescine, agmatine and spermidine uptake is shown in Figure 5. In the case of hOCT2-A, acidic amino acid residues after α-helix 9 are located outside the cell surface, so that uptake activities of the three amines were not observed. Relative positions of five functional amino acid residues necessary for transport of the three amines (red) and two amino acid residues for putrescine and spermidine (blue) are shown on α-helices 1 to 12, whose relative positions were constructed based on the model structure of hOCT2 [31]. As shown in Figure 6A and 6B, these seven amino acid residues (five red and two blue amino acid residues) are located close to one another in α-helices 9 to 12, supporting an idea that the three amines are prominently recognized by these acidic amino acid residues. Only two acidic amino acid residues (Glu^494^ and Glu^527^) in α-helices 9 to 12 did not influence uptake of the three amines. A view of the model of spermidine binding to hOCT2 from the cytoplasmic aspect of the protein is shown in Figure 6B, and a side view of the model, with the cytoplasmic aspect of the protein directed toward the bottom, is shown in Figure 6C and 6D. The binding pattern of putrescine to hOCT2 was also similar (data not shown). From these models, it can be proposed that α-helices 10 and 11 are most strongly involved in the recognition of three amines. α-Helices 9 and 12 are probably involved in polyamine and agmatine transport through the structural change of hOCT2 during their transport.

## Discussion

In mammalian cells, spermidine uptake by human carnitine transporter SLC22A16 [20], putrescine and agmatine uptake by hOCT2 [21], spermidine uptake by mouse OCT2 [22], and agmatine uptake by hMATE1 [21] have been previously reported. Although agmatine is a diamine like putrescine, it inhibited cell growth by inhibition of ornithine decarboxylase (ODC), a rate limiting enzyme of polyamine biosynthesis, through the stimulation of synthesis of antizyme [36], which is not only an inhibitor of ODC but also accelerates the degradation of ODC [37]; i.e. agmatine has an anti-polyamine effect on cell growth [36]. Thus, in this study, putrescine, spermidine and agmatine uptake by hOCTs and hMATEs, normally found in the kidney, were systematically studied. These proteins are thought to be expressed in many tissues [35].

Our results confirmed that hOCT2 catalyzes putrescine, agmatine and spermidine uptake. The uptake of putrescine and agmatine by hOCT2 was slightly more efficient than that of spermidine. Agmatine uptake was also catalyzed by hOCT2, hOCT3 and hMATE1. However, no significant amounts of agmatine were detected in any organs of mice, which were fed by normal diet (data not shown). Only a few kinds of food and drink contain agmatine [38]. Thus, hOCT2 functions as putrescine and spermidine transporter *in vivo*.

Putrescine uptake by hOCT2 in HEK293 cells has been recently reported [21]. The *Km* and *Vmax* values reported [21] were similar to values reported here. As for spermidine uptake by the carnitine transporter SLC22A16, the uptake activity in human colon carcinoma HCT116 cells (*Vmax* = 23.1 pmol/10^7^ cells/h) seems to be lower compared to the spermidine uptake activity by hOCT2, although the *Km* value is very low (0.35 µM) [20]. It has been also reported that mOCT2 can catalyze spermidine uptake with the *Km* value of 1.04 mM [22], which is low compared with the *Km* value (6.76 mM) in this study. The *Vmax* value could not be directly compared because spermidine uptake was measured using mOCT2-expressing oocytes [22]. Experiments are in progress to clarify this difference.

With regard to the active site of organic cation transporters, it has been reported that Asp^475^ on the helix 11 of rat OCT1 [39], Cys^451^ and Cys^474^ on the α-helices 10 and 11 of hOCT2 [40], and Glu^447^ on the α-helix 10 of rabbit OCT2 [30] are strongly involved in uptake of a cationic compound, TEA (tetraethylammonium). It was found that Asp^427^, Glu^448^, Glu^456^, Asp^475^ and Glu^516^ on α-helices 9, 10, 11 and 12 were strongly involved in the uptake of putrescine, agmatine and spermidine. In addition, Glu^524^ and Glu^530^ were involved in putrescine and spermidine uptake activity, and Glu^528^ and Glu^540^ were weakly involved in putrescine uptake activity. Furthermore, Asp^551^ was necessary for recognition of spermidine. In the case of PotE, a putrescine-ornithine antiporter in *E. coli* [3], four α-helices among 12 α-helices were mainly involved in the recognition of putrescine [41]. Similarly, four α-helices 9 to 12 were also involved in recognition of the three amines containing a diaminobutane moiety. It is of interest that more acidic amino acid residues were involved in the recognition of putrescine and spermidine than agmatine. This may be explained by the fact that the guanidino group in agmatine was more interactive than the primary amino- or secondary amine-group in putrescine and spermidine. Our results indicate that the COOH side of hOCT2 constitutes the active site, and the functional amino acid residues were localized to recognize the three amines with similar affinity. As for the interaction between spermidine and hOCT2, only one interaction between the primary amino-group of spermidine and Asp^475^ in hOCT2 is strong among three primary amino- and secondary amine-group (2.96 Å shown in Figure 6D). However, in case of PotD (*Kd* for spermidine: 3.2 µM), a spermidine binding protein in spermidine preferential uptake system in *E. coli* [6], all three interactions between the primary amino- or secondary amine-group of spermidine, and Glu^36^, Glu^171^ and Asp^257^ in PotD were strong (within 3 Å). The high *Km* value for spermidine may be explained by this weak interaction between spermidine and hOCT2.

It was estimated that homology of amino acid sequences between hOCT1 and hOCT2, and between hOCT2 and hOCT3 were 71% and 51%, respectively. However, agmatine uptake activity was only observed in hOCT2 and hOCT3, and putrescine and spermidine uptake was only observed in hOCT2. Thus, the location of acidic amino acid residues in the COOH side of each cation transporter is important for determining their substrate specificity.

It is known that putrescine and spermidine content in serum is normally very low (approximately 130–180 nM) [42]. When HEK293 cells transfected with pCMV6-XL4/hOCT2 were incubated with 0.3 µM spermidine, approximately 25 pmol spermidine/mg protein accumulated in cells during incubation at 37°C for 1 h. This corresponds to approximately 5 µM spermidine accumulation in cells, since the intracellular water space of mammalian cells is assumed to be 5.5 µl of cell volume/mg protein [43]. If expression of hOCT2 from pCMV6-XL4/hOCT2 is supposed to be 5-fold higher than that from genomic DNA, it is estimated that 10 to 25 µM putrescine or spermidine is accumulated in cells per day by chromosomally-encoded hOCT2. Thus, polyamine uptake by hOCT2 may contribute slightly, but significantly, to the maintenance of polyamine content in cells.

## Supporting Information

Table S1 **List of oligonucleotide primers used for mutagenesis.**
(PDF)
