# Supplementary material for: Identification of Functional Amino Acid Residues Involved in Polyamine and Agmatine Transport by Human Organic Cation Transporter 2
Source: PLoS One. 2014 Jul 14;9(7):e102234. doi: 10.1371/journal.pone.0102234 (PMC4096761; doi:10.1371/journal.pone.0102234)
Supplement: Table S1 — List of oligonucleotide primers used for mutagenesis. (PDF) [file pone.0102234.s001.pdf]

Table S1. List of oligonucleotide primers used for mutagenesis

Primer	Sequence	Number of nucleotides
hOCT2 D374N F	5'-GGCCTTGCAGGTAACAATATCTACCTG-3'	27
hOCT2 D374N R	5'-CAGGTAGATATTGTTACCTGCAAGGCC-3'	27
hOCT2 D427N F	5'-CAGTTTTTATACCTGGTAATCTACAATGGC-3'	30
hOCT2 D427N R	5'-GCCATTGTAGATTACCAGGTATAAAAACTG-3'	30
hOCT2 E448Q F	5'-ACAATGGCCTATCAGATAGTCTGCCTGGTC-3'	30
hOCT2 E448Q R	5'-ACCAGGCAGACTATCTGATAGGCCATTGTG-3'	30
hOCT2 E456Q F	5'-TGCCTGGTCAATGCTCAGCTGTACCCCAC-3'	29
hOCT2 E456Q R	5'-ATGTGGGGTACAGCTGAGCATTGACCAGGC-3'	30
hOCT2 D475N F	5'-TCCTCAATGTGTAACATTGGTGGCATCATC-3'	30
hOCT2 D475N R	5'-TGATGCCACCAATGTTACACATTGAGGAAC-3'	30
hOCT2 E494Q F	5'-CTAACATCTGGCTTCAGCTCCCGCTGATGG-3'	30
hOCT2 E494Q R	5'-CCATCAGCGGGAGCTGAAGCCAGATGTTAG-3'	30
hOCT2 E516Q F	5'-TGCTGTTGCTTCCACAGACTAAAGGGAAAG-3'	30
hOCT2 E516Q R	5'-GCTTTCCCTTTAGTCTGTGGAAGCAACAGC-3'	30
hOCT2 E524Q F	5'-GGAAAGCTTTGCCTCAGACCATCGAGGAAG-3'	30
hOCT2 E524Q R	5'-CTTCCTCGATGGTCTGAGGCAAAGCTTTCC-3'	30
hOCT2 E527Q F	5'-CCTGAGACCATCCAGGAAGCCGAAAATATG-3'	30
hOCT2 E527Q R	5'-CATATTTTCGGCTTCCTGGATGGTCTCAGG-3'	30
hOCT2 E528Q F	5'-CCTGAGACCATCGAGCAGGCCGAAAATATG-3'	30
hOCT2 E528Q R	5'-CATATTTTCGGCCTGCTCGATGGTCTCAGG-3'	30
hOCT2 E530Q F	5'-CCATCGAGGAAGCCCAGAATATGCAAAGAC-3'	30
hOCT2 E530Q R	5'-GTCTTTGCATATTCTGGGCTTCCTCGATGG-3'	30
hOCT2 E540Q F	5'-GACCAAGAAAAAATAAACAGAAGATGATTTACCTCC-3'	36
hOCT2 E540Q R	5'-GGAGGTAAATCATCTTCTGTTTATTTTTTCTTGGTC-3'	36
hOCT2 D551N F	5'-GTTCAGAAACTAAACATTCCATTGAACTCTAGAGGG-3'	36
hOCT2 D551N R	5'-CCCTCTAGAGTTCAATGGAATGTTTAGTTTCTGAAC-3'	36
